# The genome sequence of the Peppered Grey, *Eudonia truncicolella *(Stainton, 1849)

**DOI:** 10.12688/wellcomeopenres.20680.1

**Published:** 2024-02-19

**Authors:** David C. Lees, Natural History Museum Genome Acquisition Lab, Darwin Tree of Life Barcoding collective, Wellcome Sanger Institute Tree of Life Management, Samples and Laboratory team, Wellcome Sanger Institute Scientific Operations: Sequencing Operations, Wellcome Sanger Institute Tree of Life Core Informatics team, Tree of Life Core Informatics collective, Darwin Tree of Life Consortium

**Affiliations:** 1 Natural History Museum, London, England, UK

**Keywords:** Eudonia truncicolella, Peppered Grey, genome sequence, chromosomal, Lepidoptera

## Abstract

We present a genome assembly from an individual male *Eudonia truncicolella* (the Peppered Grey; Arthropoda; Insecta; Lepidoptera; Crambidae). The genome sequence is 499.1 megabases in span. Most of the assembly is scaffolded into 30 chromosomal pseudomolecules, including the Z sex chromosome. The mitochondrial genome has also been assembled and is 15.38 kilobases in length.

## Species taxonomy

Eukaryota; Metazoa; Eumetazoa; Bilateria; Protostomia; Ecdysozoa; Panarthropoda; Arthropoda; Mandibulata; Pancrustacea; Hexapoda; Insecta; Dicondylia; Pterygota; Neoptera; Endopterygota; Amphiesmenoptera; Lepidoptera; Glossata; Neolepidoptera; Heteroneura; Ditrysia; Obtectomera; Pyraloidea; Crambidae; Scopariinae; *Eudonia*; *Eudonia truncicolella* (Stainton, 1849) (NCBI:txid753179).

## Background

The Peppered Grey (otherwise commonly known as the Ground-moss Grey: Parsons & Clancy, 2023), *Eudonia truncicolella* (Stainton, 1849), is a greyish scopariine pyralid moth with a mottled appearance and roughly scaled darker markings, measuring about 18–23 mm in wingspan ( Goater *et al.*, 1986) and 9–11 mm in length ( Lewis, 2023). It is superficially similar to several scopariine species, others of which exhibit a rather X-shaped dark reniform stigma and in the United Kingdom is particularly similar to the Moorland Grey *E. murana* (Curtis, 1827), lacking in Southern England and Ireland, and from which it can reliably be separated by dissection. The dark postmedian line of the forewing has a more indented appearance than *E. murana*, but the forewing of that species has a sclerotised hook on the underside in addition to the usual coupling system ( Parsons & Clancy, 2023: 302). It is also quite similar to *Scoparia ambigualis* (Treitschke, 1829) whose forewing has greyer markings and a yellowish hue ( Parsons & Clancy, 2023). For an online guide to British species see Lewis (2023). However, the female genitalia are similar to those of *E. lacustrata* (Panzer, 1804), and elsewhere in the Palaearctic (China), the male genitalia are similar to those of *E. wolongensis* ( Li *et al.*, 2012), and those of both sexes (see Lepiforum, 2023; Parsons & Clancy, 2023: 467, 478) similar to *E. apicifusca* Sasaki, 1999 ( Li *et al.*, 2012), so DNA barcoding is also helpful for verification.

The Peppered Grey is widespread in woodland in the British Isles, also occurring in heathland and gardens and is locally common, ranging northwards as far as the Inner Hebrides and Orkney, and westwards to Isle of Man and Ireland ( Goater *et al.*, 1986; Parsons & Clancy, 2023). It is also very widespread in the Palaearctic from western Ireland and Spain and northern Scandinavia as far east as China and Japan ( GBIF Secretariat, 2023; Li *et al.*, 2012).

The adult moth flies in mid-June to October (mostly in July and August) in Britain, resting on tree trunks by day from which it is very flighty, and after dusk is recorded to visit the flowers of Field Scabious *Knautia arvensis* ( Parsons & Clancy, 2023). The dark brownish green larva with a dorsal dark line flanked by rather quadrate spots and lateral crescentic spots on each segment ( Lepiforum, 2023; Parsons & Clancy, 2023: 312) feeds from September to June in silken galleries under moss such as *Hypnum cupressiforme* ( Lepiforum, 2023), *Dicranum scoparium*, and *Campylotus* sp. ( Parsons & Clancy, 2023) or sometimes under stones ( Goater *et al.*, 1986). It usually pupates as an orange-brown pupa in moss, from June to July ( Goater *et al.*, 1986).

DNA barcode sequences on BOLD (30/11/2023) belong to a single BIN cluster, BOLD:AAB1558 with up to about 1.28% intraspecific divergence and its two nearest neighbours on BOLD exhibit from about 2.8–4% pairwise divergence; these are *Eudonia* “sp. 1 WL-2017” from China (BOLD:AED8759) and then *Scoparia exhibitalis* Walker, [1866] from Australia (BOLD:AAC3288). *E. truncicolella* was treated in a phylogeny by Léger *et al.* (2019) based on five nuclear genes and one mitochondrial gene and belongs to a clade comprising *Eudonia lacustrata* and *E. mercurella* (L., 1758) among the described species treated there.

The genome of the Peppered Grey, *Eudonia truncicolella*, was sequenced as part of the Darwin Tree of Life Project, a collaborative effort to sequence all named eukaryotic species in the Atlantic Archipelago of Britain and Ireland. Here we present a chromosomally complete genome sequence for *Eudonia truncicolella*, based on one male specimen from Beinn Eighe National Nature Reserve, Scotland.

## Genome sequence report

**Figure 1.**
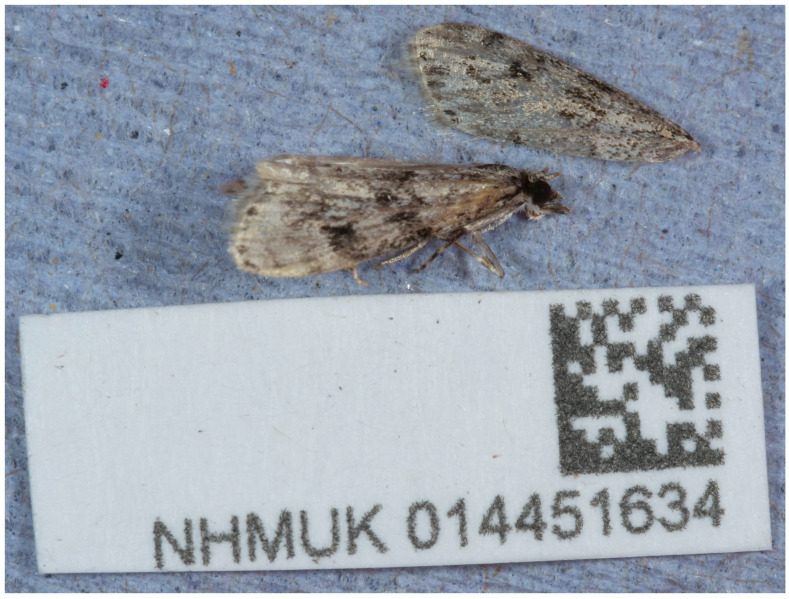
Photograph of the *Eudonia truncicolella* (ilEudTrun2) specimen used for genome sequencing.

The genome was sequenced from one male *Eudonia truncicolella* ( Figure 1) collected from Beinn Eighe National Nature Reserve (see Methods). A total of 51-fold coverage in Pacific Biosciences single-molecule HiFi long reads was generated. Primary assembly contigs were scaffolded with chromosome conformation Hi-C data. Manual assembly curation corrected 5 missing joins or mis-joins and removed one haplotypic duplication, increasing the scaffold count by 1.

**Figure 2.**
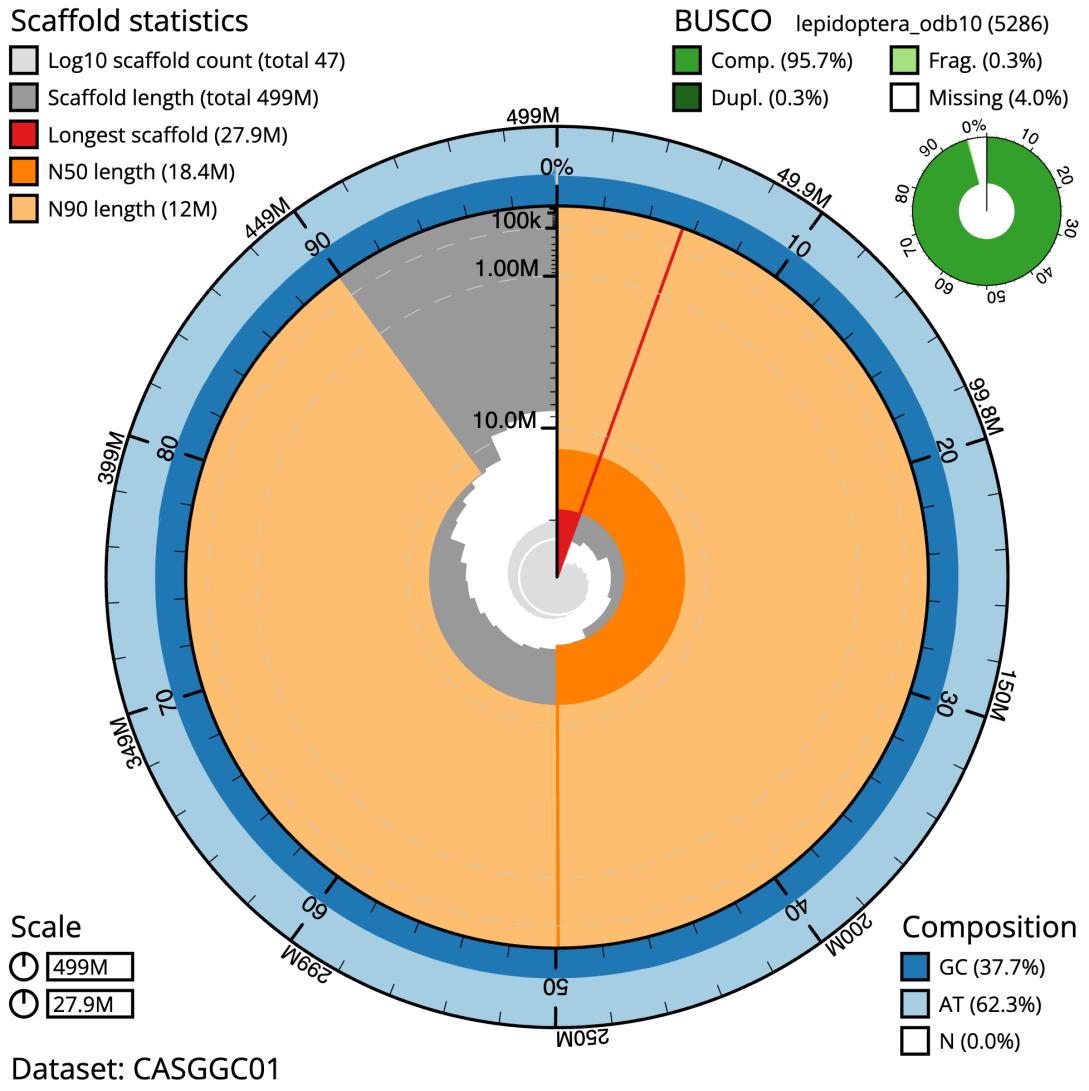
Genome assembly of *Eudonia truncicolella*, ilEudTrun2.1: metrics. The BlobToolKit Snailplot shows N50 metrics and BUSCO gene completeness. The main plot is divided into 1,000 size-ordered bins around the circumference with each bin representing 0.1% of the 499,132,009 bp assembly. The distribution of scaffold lengths is shown in dark grey with the plot radius scaled to the longest scaffold present in the assembly (27,890,741 bp, shown in red). Orange and pale-orange arcs show the N50 and N90 scaffold lengths (18,437,561 and 11,974,810 bp), respectively. The pale grey spiral shows the cumulative scaffold count on a log scale with white scale lines showing successive orders of magnitude. The blue and pale-blue area around the outside of the plot shows the distribution of GC, AT and N percentages in the same bins as the inner plot. A summary of complete, fragmented, duplicated and missing BUSCO genes in the lepidoptera_odb10 set is shown in the top right. An interactive version of this figure is available at https://blobtoolkit.genomehubs.org/view/Eudonia%20truncicolella/dataset/CASGGC01/snail.

**Figure 3.**
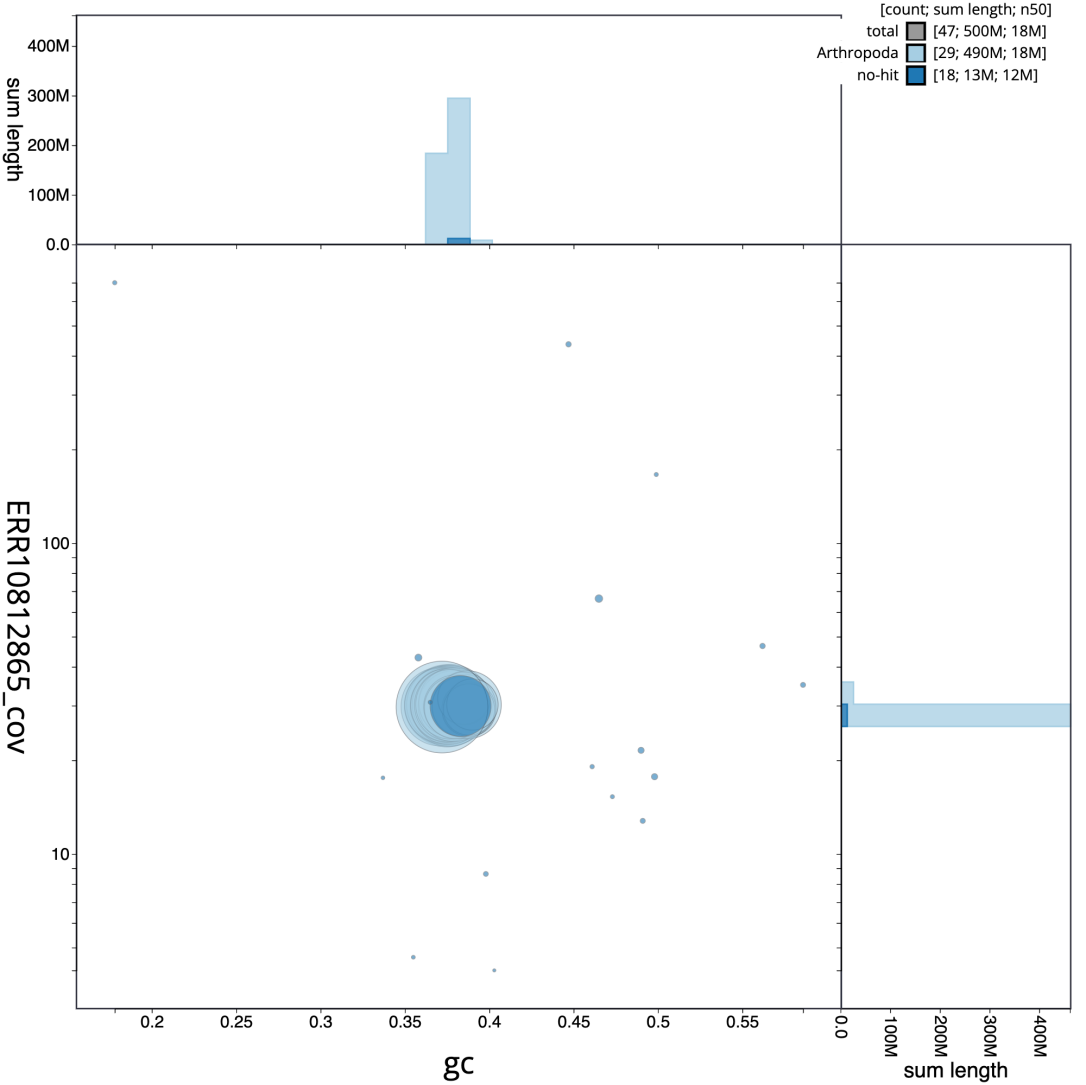
Genome assembly of *Eudonia truncicolella*, ilEudTrun2.1: BlobToolKit GC-coverage plot. Scaffolds are coloured by phylum. Circles are sized in proportion to scaffold length. Histograms show the distribution of scaffold length sum along each axis. An interactive version of this figure is available at https://blobtoolkit.genomehubs.org/view/Eudonia%20truncicolella/dataset/CASGGC01/blob.

**Figure 4.**
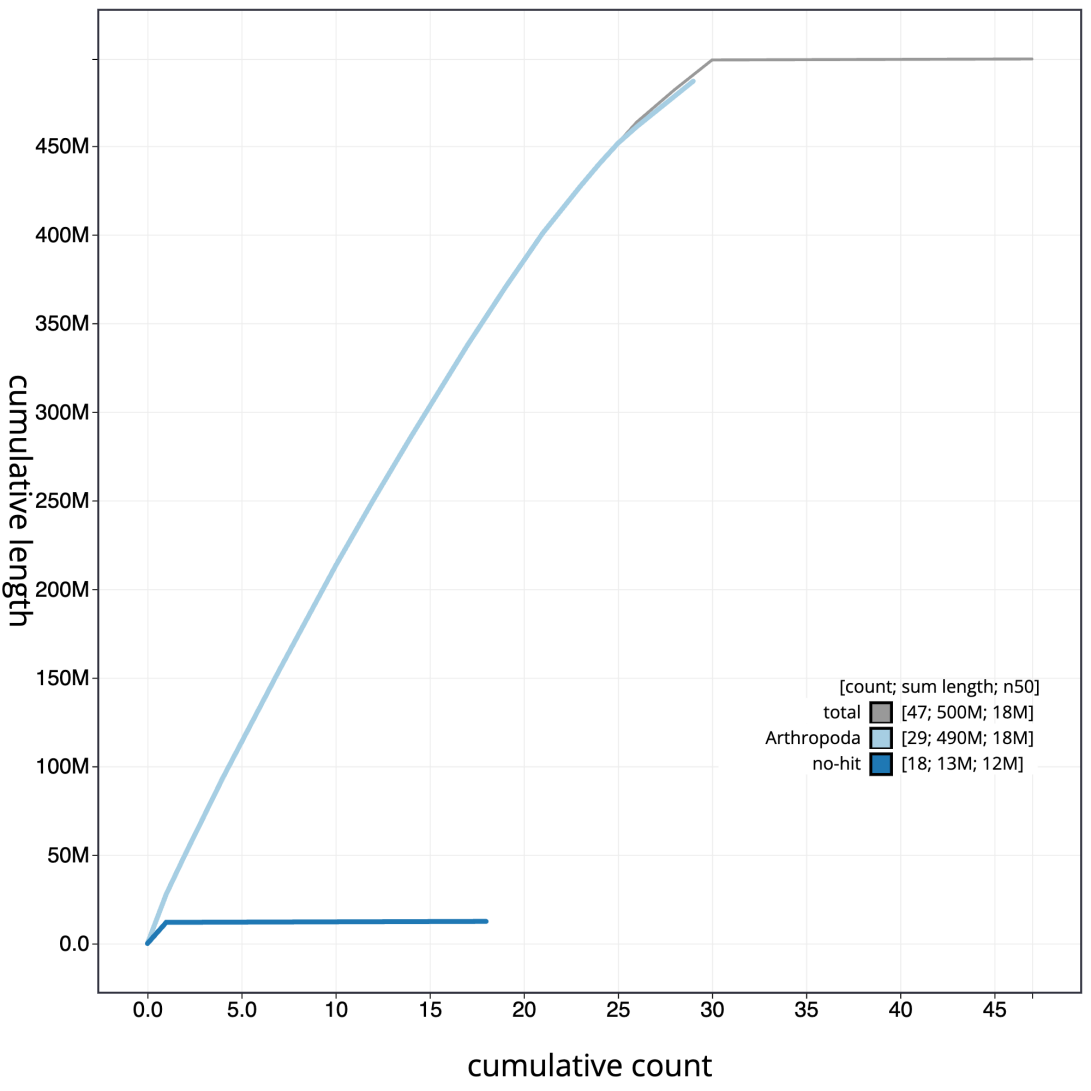
Genome assembly of *Eudonia truncicolella*, ilEudTrun2.1: BlobToolKit cumulative sequence plot. The grey line shows cumulative length for all scaffolds. Coloured lines show cumulative lengths of scaffolds assigned to each phylum using the buscogenes taxrule. An interactive version of this figure is available at https://blobtoolkit.genomehubs.org/view/Eudonia%20truncicolella/dataset/CASGGC01/cumulative.

**Figure 5.**
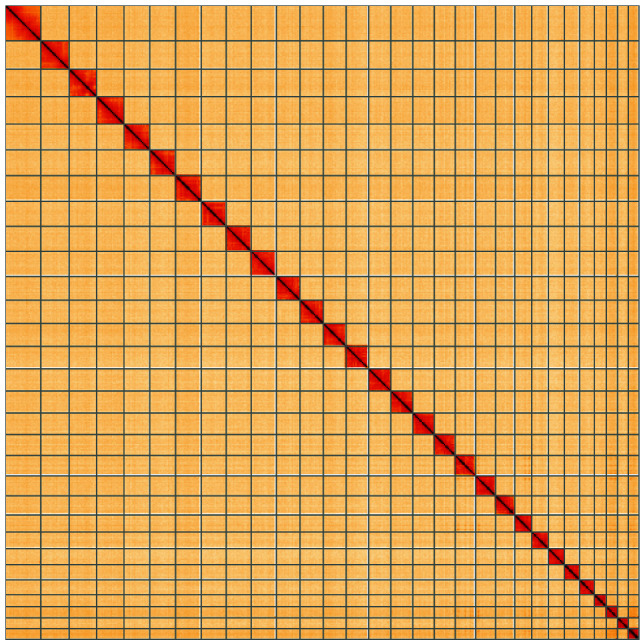
Genome assembly of *Eudonia truncicolella*, ilEudTrun2.1: Hi-C contact map of the ilEudTrun2.1 assembly, visualised using HiGlass. Chromosomes are shown in order of size from left to right and top to bottom. An interactive version of this figure may be viewed at https://genome-note-higlass.tol.sanger.ac.uk/l/?d=aWzNX_veSgmZ678sikdPvw.

The final assembly has a total length of 499.1 Mb in 46 sequence scaffolds with a scaffold N50 of 18.4 Mb ( Table 1). The snailplot in Figure 2 provides a summary of the assembly statistics, while the distribution of assembly scaffolds on GC proportion and coverage is shown in Figure 3. The cumulative assembly plot in Figure 4 shows curves for subsets of scaffolds assigned to different phyla. Most (99.89%) of the assembly sequence was assigned to 30 chromosomal-level scaffolds, representing 29 autosomes and the Z sex chromosome. The Z chromosome was assigned by alignment to that of *Eudonia lacustrata* (GCA_947562085.1) ( Boyes *et al.*, 2023). Chromosome-scale scaffolds confirmed by the Hi-C data are named in order of size ( Figure 5; Table 2). While not fully phased, the assembly deposited is of one haplotype. Contigs corresponding to the second haplotype have also been deposited. The mitochondrial genome was also assembled and can be found as a contig within the multifasta file of the genome submission.

**Table 1.** Genome data for *Eudonia truncicolella*, ilEudTrun2.1.

Project accession data		
Assembly identifier	ilEudTrun2.1	
Species	*Eudonia truncicolella*	
Specimen	ilEudTrun2	
NCBI taxonomy ID	753179	
BioProject	PRJEB59300	
BioSample ID	SAMEA14448308	
Isolate information	ilEudTrun2, abdomen (DNA sequencing), thorax (Hi-C sequencing)	
Assembly metrics *		*Benchmark*
Consensus quality (QV)	65.6	*≥ 50*
*k*-mer completeness	100%	*≥ 95%*
BUSCO **	C:95.7%[S:95.4%,D:0.3%], F:0.3%,M:4.0%,n:5,286	*C ≥ 95%*
Percentage of assembly mapped to chromosomes	99.89%	*≥ 95%*
Sex chromosomes	Z chromosome	*localised * *homologous pairs*
Organelles	Mitochondrial genome assembled	*complete single * *alleles*
Raw data accessions		
PacificBiosciences SEQUEL II	ERR10812865	
Hi-C Illumina	ERR10818322	
Genome assembly		
Assembly accession	GCA_949315975.1	
*Accession of alternate haplotype*	GCA_949315915.1	
Span (Mb)	499.1	
Number of contigs	103	
Contig N50 length (Mb)	8.9	
Number of scaffolds	46	
Scaffold N50 length (Mb)	18.4	
Longest scaffold (Mb)	27.9	

* Assembly metric benchmarks are adapted from column VGP-2020 of “Table 1: Proposed standards and metrics for defining genome assembly quality” from Rhie *et al.* (2021). ** BUSCO scores based on the lepidoptera_odb10 BUSCO set using v5.3.2. C = complete [S = single copy, D = duplicated], F = fragmented, M = missing, n = number of orthologues in comparison. A full set of BUSCO scores is available at https://blobtoolkit.genomehubs.org/view/Eudonia%20truncicolella/dataset/CASGGC01/busco.

**Table 2.** Chromosomal pseudomolecules in the genome assembly of *Eudonia truncicolella*, ilEudTrun2.

INSDC accession	Chromosome	Length (Mb)	GC%
OX438826.1	1	22.31	37.5
OX438827.1	2	21.61	37.5
OX438828.1	3	21.58	37.5
OX438829.1	4	20.27	37.5
OX438830.1	5	20.26	37.0
OX438831.1	6	20.23	37.5
OX438832.1	7	19.69	37.5
OX438833.1	8	19.68	37.5
OX438834.1	9	19.68	37.5
OX438835.1	10	18.6	37.5
OX438836.1	11	18.44	37.0
OX438837.1	12	17.97	37.5
OX438838.1	13	17.7	37.5
OX438839.1	14	17.33	37.5
OX438840.1	15	17.33	37.5
OX438841.1	16	16.98	37.5
OX438842.1	17	16.49	38.0
OX438843.1	18	15.94	38.0
OX438844.1	19	15.76	37.5
OX438845.1	20	15.07	38.5
OX438846.1	21	13.34	38.5
OX438847.1	22	13.11	38.0
OX438848.1	23	12.57	38.0
OX438849.1	24	11.97	38.5
OX438850.1	25	11.72	38.5
OX438851.1	26	9.37	39.0
OX438852.1	27	8.81	38.5
OX438853.1	28	8.43	39.0
OX438854.1	29	8.43	39.0
OX438825.1	Z	27.89	37.0
OX438855.1	MT	0.02	18.0

The estimated Quality Value (QV) of the final assembly is 65.6 with *k*-mer completeness of 100%, and the assembly has a BUSCO v5.3.2 completeness of 95.7% (single = 95.4%, duplicated = 0.3%), using the lepidoptera_odb10 reference set ( *n* = 5,286).

Metadata for specimens, barcode results, spectra estimates, sequencing runs, contaminants and pre-curation assembly statistics are given at https://links.tol.sanger.ac.uk/species/753179.

## Methods

### Sample acquisition and nucleic acid extraction

A male *Eudonia truncicolella* (specimen ID NHMUK014451634, ToLID ilEudTrun2) was collected from Beinn Eighe National Nature Reserve, Scotland, UK (latitude 57.63, longitude –5.35) on 2021-09-10 using a light trap. The specimen was collected and identified by David Lees (Natural History Museum), and then preserved by dry freezing at –80 °C.

High molecular weight (HMW) DNA was extracted at the Tree of Life laboratory, Wellcome Sanger Institute (WSI), following a sequence of core procedures: sample preparation; sample homogenisation; HMW DNA extraction; DNA fragmentation; and DNA clean-up. The ilEudTrun2 sample was weighed and dissected on dry ice ( Jay *et al.*, 2023). Abdomen tissue from the ilEudTrun2 sample was homogenised using a PowerMasher II tissue disruptor ( Denton *et al.*, 2023a). HMW DNA was extracted using the Automated MagAttract v1 protocol ( Sheerin *et al.*, 2023). The DNA was sheared into an average fragment size of 12–20 kb in a Megaruptor 3 system with speed setting 30 ( Todorovic *et al.*, 2023). Sheared DNA was purified by solid-phase reversible immobilisation ( Strickland *et al.*, 2023): in brief, the method employs a 1.8X ratio of AMPure PB beads to sample to eliminate shorter fragments and concentrate the DNA. The concentration of the sheared and purified DNA was assessed using a Nanodrop spectrophotometer and Qubit Fluorometer and Qubit dsDNA High Sensitivity Assay kit. Fragment size distribution was evaluated by running the sample on the FemtoPulse system.

Protocols developed by the WSI Tree of Life laboratory are publicly available on protocols.io ( Denton *et al.*, 2023b).

### Sequencing

Pacific Biosciences HiFi circular consensus DNA sequencing libraries were constructed according to the manufacturers’ instructions. DNA sequencing was performed by the Scientific Operations core at the WSI on a Pacific Biosciences SEQUEL II instrument. Hi-C data were also generated from thorax tissue of ilEudTrun2 using the Arima2 kit and sequenced on the Illumina NovaSeq 6000 instrument.

### Genome assembly, curation and evaluation

Assembly was carried out with Hifiasm ( Cheng *et al.*, 2021) and haplotypic duplication was identified and removed with purge_dups ( Guan *et al.*, 2020). The assembly was then scaffolded with Hi-C data ( Rao *et al.*, 2014) using YaHS ( Zhou *et al.*, 2023). The assembly was checked for contamination and corrected as described previously ( Howe *et al.*, 2021). Manual curation was performed using HiGlass ( Kerpedjiev *et al.*, 2018) and Pretext ( Harry, 2022). The mitochondrial genome was assembled using MitoHiFi ( Uliano-Silva *et al.*, 2023), which runs MitoFinder ( Allio *et al.*, 2020) or MITOS ( Bernt *et al.*, 2013) and uses these annotations to select the final mitochondrial contig and to ensure the general quality of the sequence.

A Hi-C map for the final assembly was produced using bwa-mem2 ( Vasimuddin *et al.*, 2019) in the Cooler file format ( Abdennur & Mirny, 2020). To assess the assembly metrics, the *k*-mer completeness and QV consensus quality values were calculated in Merqury ( Rhie *et al.*, 2020). This work was done using Nextflow ( Di Tommaso *et al.*, 2017) DSL2 pipelines “sanger-tol/readmapping” ( Surana *et al.*, 2023a) and “sanger-tol/genomenote” ( Surana *et al.*, 2023b). The genome was analysed within the BlobToolKit environment ( Challis *et al.*, 2020) and BUSCO scores ( Manni *et al.*, 2021; Simão *et al.*, 2015) were calculated.

Table 3 contains a list of relevant software tool versions and sources.

**Table 3.** Software tools: versions and sources.

Software tool	Version	Source
BlobToolKit	4.1.7	https://github.com/blobtoolkit/ blobtoolkit
BUSCO	5.3.2	https://gitlab.com/ezlab/busco
Hifiasm	0.16.1-r375	https://github.com/chhylp123/ hifiasm
HiGlass	1.11.6	https://github.com/higlass/higlass
Merqury	MerquryFK	https://github.com/thegenemyers/ MERQURY.FK
MitoHiFi	2	https://github.com/marcelauliano/ MitoHiFi
PretextView	0.2	https://github.com/wtsi-hpag/ PretextView
purge_dups	1.2.3	https://github.com/dfguan/purge_ dups
sanger-tol/ genomenote	v1.0	https://github.com/sanger-tol/ genomenote
sanger-tol/ readmapping	1.1.0	https://github.com/sanger-tol/ readmapping/tree/1.1.0
YaHS	1.2a	https://github.com/c-zhou/yahs

### Wellcome Sanger Institute – Legal and Governance

The materials that have contributed to this genome note have been supplied by a Darwin Tree of Life Partner. The submission of materials by a Darwin Tree of Life Partner is subject to the **‘Darwin Tree of Life Project Sampling Code of Practice’**, which can be found in full on the Darwin Tree of Life website here. By agreeing with and signing up to the Sampling Code of Practice, the Darwin Tree of Life Partner agrees they will meet the legal and ethical requirements and standards set out within this document in respect of all samples acquired for, and supplied to, the Darwin Tree of Life Project.

Further, the Wellcome Sanger Institute employs a process whereby due diligence is carried out proportionate to the nature of the materials themselves, and the circumstances under which they have been/are to be collected and provided for use. The purpose of this is to address and mitigate any potential legal and/or ethical implications of receipt and use of the materials as part of the research project, and to ensure that in doing so we align with best practice wherever possible. The overarching areas of consideration are:

•   Ethical review of provenance and sourcing of the material

•   Legality of collection, transfer and use (national and international)

Each transfer of samples is further undertaken according to a Research Collaboration Agreement or Material Transfer Agreement entered into by the Darwin Tree of Life Partner, Genome Research Limited (operating as the Wellcome Sanger Institute), and in some circumstances other Darwin Tree of Life collaborators.

## Data Availability

European Nucleotide Archive: *Eudonia truncicolella*. Accession number PRJEB59300; https://identifiers.org/ena.embl/PRJEB59300 ( Wellcome Sanger Institute, 2023). The genome sequence is released openly for reuse. The *Eudonia truncicolella* genome sequencing initiative is part of the Darwin Tree of Life (DToL) project. All raw sequence data and the assembly have been deposited in INSDC databases. The genome will be annotated using available RNA-Seq data and presented through the Ensembl pipeline at the European Bioinformatics Institute. Raw data and assembly accession identifiers are reported in Table 1.

## Author information

Members of the Natural History Museum Genome Acquisition Lab are listed here: https://doi.org/10.5281/zenodo.7139035.

Members of the Darwin Tree of Life Barcoding collective are listed here: https://doi.org/10.5281/zenodo.4893703.

Members of the Wellcome Sanger Institute Tree of Life Management, Samples and Laboratory team are listed here: https://doi.org/10.5281/zenodo.10066175.

Members of Wellcome Sanger Institute Scientific Operations: Sequencing Operations are listed here: https://doi.org/10.5281/zenodo.10043364.

Members of the Wellcome Sanger Institute Tree of Life Core Informatics team are listed here: https://doi.org/10.5281/zenodo.10066637.

Members of the Tree of Life Core Informatics collective are listed here: https://doi.org/10.5281/zenodo.5013541.

Members of the Darwin Tree of Life Consortium are listed here: https://doi.org/10.5281/zenodo.4783558.
