# Supplementary material for: The genome sequence of the Peppered Grey, Eudonia truncicolella (Stainton, 1849)
Source: Wellcome Open Res. 2024 Feb 19;9:44. [Version 1] doi: 10.12688/wellcomeopenres.20680.1 (PMC11126902; doi:10.12688/wellcomeopenres.20680.1)
Supplement: Peer review history

# Reviewer report: Reviewer response for version 1

**DOI:** 10.21956/wellcomeopenres.22885.r81422

**Published:** 2024-05-24

**Authors:** Yasuko Akiyama-Oda, Koh Nakagawa

**Affiliations:** 1 JT Biohistory Research Hall, Takatsuki, Osaka, Japan

The genome note submitted by Lees et al. reports the genome sequence of a moth, the peppered grey *Eudonia truncicolella*. Overall, I found the note to be well-written, with interactive versions of the figures provided for enhanced clarity. I only have a few minor suggestions:

 One aspect that could improve understanding would be to provide information on the chromosome count, since most of the assembly sequences were assigned to 30 chromosome-level scaffolds.

 Additionally, I suggest including information about the extent of differences between the two haplotypes deposited in the data. This would be valuable for future study of inter- and intra-species differences.

 Lastly, it would be informative to mention the similarity between the Z chromosome of *E*. *truncicolellai *and that of *E*. *lacustrata*, considering that the Z chromosome was assigned using the sequence of the latter.

 The following is a repot from the co-reviewer:

 I am impressed by the thorough genome data acquisition protocol and the use of the latest and most suitable tools for assembly, data processing, and evaluation. However, to make the research outcomes more widely usable by researchers specializing in moss and other insects, I have a few minor comments.

 1. The significance of *Eudonia truncicolella* as a subject for molecular research should be described [in Background].

 Although the taxonomy, distribution, and ecological knowledge of *E. truncicolella* are comprehensively covered in the Background, if there have been molecular studies using this or closely related species, it would be beneficial to include details of such studies. This would help convey the significance of sequencing the genome of this species more effectively to the readers.

 2. From Background, it is clear that *E. truncicolella* often has morphological similarities with closely related species, making it difficult to fully cover the classification system based solely on morphological characteristics. In addition to the phrase "DNA barcoding is also helpful for verification," it would be helpful to add a sentence emphasizing the utility of DNA barcoding.

 For example, a statement like "Combining morphological species taxonomy with DNA barcoding establishes the classification system for this species" could be added.

 3. The reference to BOLD should include a citation unless there is a specific reason not to [in Background]. see [Ref 1]

 4. The method of species identification for the specimen should be briefly supplemented [in Methods].

 It should be briefly described whether the species identification was based on body markings, genitalia morphology, or another morphological comparison, or a combination of these factors.

Are sufficient details of methods and materials provided to allow replication by others?

Yes

Is the rationale for creating the dataset(s) clearly described?

Yes

Are the datasets clearly presented in a useable and accessible format?

Yes

Are the protocols appropriate and is the work technically sound?

Yes

Reviewer Expertise:

Developmental biology, Transcriptomics, Genomics

We confirm that we have read this submission and believe that we have an appropriate level of expertise to confirm that it is of an acceptable scientific standard.

## References

[1] : bold: The Barcode of Life Data System (http://www.barcodinglife.org). *Mol Ecol Notes* .2007;7(3) : 10.1111/j.1471-8286.2007.01678.x 355-364 10.1111/j.1471-8286.2007.01678.x 18784790 PMC1890991

# Reviewer report: Reviewer response for version 1

**DOI:** 10.21956/wellcomeopenres.22885.r81418

**Published:** 2024-05-22

**Authors:** Thayná Lopes

**Affiliations:** 1 Laboratório de Citogenética e Entomologia Molecular, Universidade Estadual de Londrina, Londrina, State of Paraná, Brazil

This genome note reports a high-quality assembly and annotation of the genome of the Peppered Grey, *Eudonia truncicolella*.

 The background section is well written, and there is also plenty of species information, which adds to the data note.

 The methods used were appropriate for this assembly, and the resultant genome is of high quality and well validated. The data will be an invaluable resource for moth researchers in genomics and entomology.

Are sufficient details of methods and materials provided to allow replication by others?

Yes

Is the rationale for creating the dataset(s) clearly described?

Yes

Are the datasets clearly presented in a useable and accessible format?

Yes

Are the protocols appropriate and is the work technically sound?

Yes

Reviewer Expertise:

Bioinformatics and molecular genetics of insects

I confirm that I have read this submission and believe that I have an appropriate level of expertise to confirm that it is of an acceptable scientific standard.
